# Jammed Microgel Inks for 3D Printing Applications

**DOI:** 10.1002/advs.201801076

**Published:** 2018-10-24

**Authors:** Christopher B. Highley, Kwang Hoon Song, Andrew C. Daly, Jason A. Burdick

**Affiliations:** ^1^ Department of Bioengineering University of Pennsylvania 210 South 33rd Street Philadelphia PA 19104 USA

**Keywords:** 3D printing, biomaterials, hydrogels, inks, microgels

## Abstract

3D printing involves the development of inks that exhibit the requisite properties for both printing and the intended application. In bioprinting, these inks are often hydrogels with controlled rheological properties that can be stabilized after deposition. Here, an alternate approach is developed where the ink is composed exclusively of jammed microgels, which are designed to incorporate a range of properties through microgel design (e.g., composition, size) and through the mixing of microgels. The jammed microgel inks are shear‐thinning to permit flow and rapidly recover upon deposition, including on surfaces or when deposited in 3D within hydrogel supports, and can be further stabilized with secondary cross‐linking. This platform allows the use of microgels engineered from various materials (e.g., thiol‐ene cross‐linked hyaluronic acid (HA), photo‐cross‐linked poly(ethylene glycol), thermo‐sensitive agarose) and that incorporate cells, where the jamming process and printing do not decrease cell viability. The versatility of this particle‐based approach opens up numerous potential biomedical applications through the printing of a more diverse set of inks.

3D bioprinting techniques are used to organize cells and materials into 3D structures, toward applications in the modeling of functional and dysfunctional tissue systems and potentially for tissue and organ transplantation.^1^, ^2^, ^3^ There are numerous challenges to 3D bioprinting, such as the stability and resolution of printed constructs and restrictions on materials used, since they must be both nontoxic and compatible with printing processes.^3^, ^4^, ^5^, ^6^ For extrusion printing, a material must flow from a reservoir onto a print surface and then rapidly stabilize to preserve fidelity of the printed structure to the computer design.^4^, ^7^, ^8^ Soft, hydrogel materials have long been valuable in the engineering of tissues, due to their tunable biophysical and biochemical properties and their ability to encapsulate cells^9^, ^10^, ^11^, ^12^, ^13^; however, hydrogels can be challenging to print without additional modification or the use of additives.^14^, ^15^

In order to address these limitations in the printing of hydrogels, we propose here the use of jammed microgels as inks for bioprinting. Microparticles in a jammed system are densely packed and immobilized by physical interactions with surrounding particles, resulting in macroscopic materials that behave as solids until enough force is applied to induce movement.^16^, ^17^, ^18^, ^19^, ^20^ In the case of highly dense microgel formulations such as those used here, the microgel volume may deform elastically below the yield stress, but there is no movement of individual microgels. However, an application of sufficient stress to a volume of microgels results in movement of the microgels relative to one another as stress overcomes the packing forces that resist motion. Further, upon reducing an applied stress below the yield stress, the system recovers. This flow and recovery of the jammed system in response to stress meets the design demands on inks for 3D printing, without the need for changes or rearrangements in the molecular structures of the materials for printing. Additionally, the jamming of microgels should be independent of microgel composition, potentially allowing the printing of any hydrogel material that can be processed into microgels.

Granular hydrogel materials have been employed previously in biomedical applications, including as porous 3D scaffolds,^21^ as injectable hydrogel systems designed to repair tissues,^22^, ^23^ for local drug^24^ and growth factor^25^ delivery, and as support matrices for 3D printing to enable printing of complex structures.^26^, ^27^, ^28^, ^29^, ^30^ Particulate materials have been used as inks for 3D printing previously (e.g., colloids,^31^ degradable microparticles,^32^ elastomers^33^), but thus far have not included jammed microgels. These are unique in their application as bioinks in that they enable cross‐linked hydrogel particles to be formed as an aggregate bulk that can be extruded as stable filaments, without requiring the engineering of interparticle interactions, and requiring no material in the bioink beyond the particles themselves.

To fabricate microgel inks, we first used microfluidic devices (e.g., fluid focusing, T‐junctions) to form microgels, where controlled emulsions (continuous oil phase) were used to form droplets from hydrogel precursor components that were then stabilized during cross‐linking (Figure S1, Supporting Information). There is much versatility to this approach, allowing the fabrication of microgels from numerous materials, across a wide range of sizes, and incorporating biological components (e.g., cells, therapeutics).^34^, ^35^, ^36^ To illustrate this versatility, we fabricated microgels from norbornene‐modified hyaluronic acid (NorHA), poly(ethylene glycol) diacrylate (PEGDA), and agarose, where cross‐linking was performed in either the presence of a photoinitiator and light (NorHA and PEGDA) or with cooling (agarose) (**Figure** **1**a). These three microgel types were selected for their diversity, since they are formed from polymers that are both charged and uncharged to consider electrostatics and through various cross‐linking mechanisms (radical chain‐growth polymerization, thiol‐ene photoinitiated cross‐linking, and thermally‐induced physical cross‐linking). It should be noted that other techniques, such as spraying,^37^, ^38^ jetting,^39^ and ionic cross‐linking^37^, ^39^, ^40^ can also be used to fabricate microgels, depending on the material being used.

**Figure 1:**
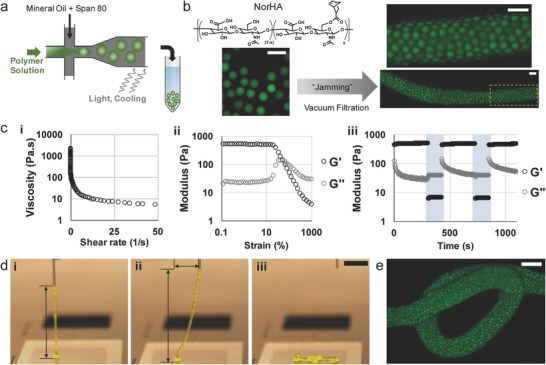
Jammed microgel ink fabrication, rheological properties, and extrusion. a) Microgel fabrication process via water‐in‐oil emulsion on a microfluidic device, where the polymer is dissolved in aqueous solution and then cross‐linked through a photoinitiated polymerization or cooling. b) Representative fluorescent images of suspended microgels (left) fabricated from 2 wt% NorHA that are jammed through vacuum filtration into a solid (right) that can be extruded from a syringe. A yellow dotted box in (b‐right, bottom) indicates acquisition position of (b‐right, top) image. c) Rheological characterization of jammed NorHA microgel inks showing (i) decreased viscosity with continuously increasing shear rates (0–50 s^−1^), (ii) shear‐yielding with increase in strain (0.037–1000%, 1 Hz), and (iii) shear‐thinning and self‐healing through low (unshaded, 1% strain, 1 Hz) and high (shaded, 500% strain, 1 Hz) strain cycles. d) Images during (i) NorHA ink extrusion, (ii) needle translation to the right, and (iii) failure of the ink. e) Fluorescent image of NorHA microgel ink after failure as shown in (d‐iii). Scale bars in (b): 200 µm, (d): 5 mm, (e): 500 µm.

NorHA microgels were formed on a microfluidic device with thiol‐ene cross‐linking, as NorHA has been used to fabricate hydrogels with tuned mechanics, controlled degradation, and encapsulated cells (Figure 1b and Figure S2, Supporting Information).^41^, ^42^ After cross‐linking, microgels were washed from the oil into buffer, and were uniform in size distribution with diameters of ≈100 µm. Microgels were then “jammed” through the removal of aqueous medium between the particles, either through centrifugation over a filter or through the use of vacuum filtration, resulting in an extrudable ink with clearly visible microgel components upon microscopic examination (Figure 1b).

The microgel inks exhibited rheological properties that are important for 3D printing. For example, the granular NorHA ink displayed shear‐thinning behavior, including decreased viscosity with increasing shear rate (Figure 1c‐i). The ink behaved as an elastic hydrogel at low strains, but then yielded at higher strains (e.g., ≈85% strain for NorHA microgels) (Figure 1c‐ii). Additionally, the microgel inks underwent a rapid, reversible transition to a liquid‐like, viscous state from a solid‐like, elastic state immediately upon application of high strain in oscillatory strain sweeps (Figure 1c‐iii). This behavior, likely due to disrupted contacts between microgels at higher strains, reflects the microgel ink's ability to flow during extrusion and rapidly stabilize after deposition, characteristic of the behavior of colloidal microgel suspensions.^20^ The pressure to drive extrusion from a syringe and through a 260 µm nozzle was similar to the pressure needed under the same conditions for extrusion of a low viscosity, liquid solution of NorHA (Figure S3, Supporting Information).

To illustrate the printing of the NorHA microgel ink, a vertical filament was printed from a glass surface with a 260 µm inner diameter nozzle. The ink flowed evenly from the nozzle, i) forming a regular filament that extended vertically 2 cm without breaking (Figure 1d‐i and Movie S1, Supporting Information), ii) resisting gravity when the needle was translated horizontally (Figure 1d‐ii and Movie S1, Supporting Information), and iii) breaking away from the nozzle after a horizontal translation exceeding 1 cm, retracting elastically and coiling to the surface (Figure 1d‐iii and Movie S1, Supporting Information). The microgel ink structure was retained after collapse (Figure 1e), indicating the stability of the jammed microgels even with elastic deformation. The filaments could then be dissociated in buffer with agitation, with no quantified difference in microgel diameter or roundness from the original microgels used for ink fabrication (Figure S4, Supporting Information).

General characteristics of the jammed microgel ink—elastic response at low strains, yielding and flow at increasing strain, shear‐thinning behavior, response to frequency changes, and the formation of stable filament structures after extrusion were similar for inks formed from PEGDA and agarose microgels, although magnitudes varied with material (**Figure** **2**a,b and Figure S5, Supporting Information). In addition, a mixture of jammed NorHA and PEGDA microgels, including both charged and uncharged components, were formulated into an ink and could be printed into filaments with cross‐sectional diameters similar to filaments formed with microgel inks composed of either NorHA or PEGDA alone (Figure 2c,d). These findings suggest that the properties important to printability are largely a function of jamming and are to some extent independent of the composition of the microgel used. This aligns with previous observations of the mechanical properties and flow behaviors of densely packed hydrogel particles at the granular (100+ µm) scale.^20^ Under low strain, these systems were described as behaving as elastic hydrogels with moduli scaling with polymer concentration and independent of particle size and molecular composition. Qualitatively, they behaved similarly, although specific flow properties were altered by molecular composition where interparticle friction may have played a role. Indeed, the interparticle interactions can be further engineered to adjust properties, such as the elastic moduli and yield strain, of densely packed microgels.

**Figure 2:**
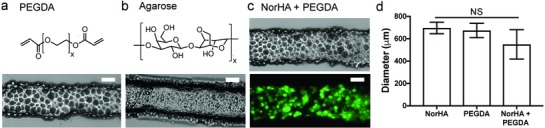
Diversity in microgel compositions for ink fabrication. a) Light microscopy image of extruded poly(ethylene glycol) (PEGDA) microgel ink fabricated from photo‐cross‐linked PEGDA (10 wt%). b) Light microscopy image of extruded agarose (1 wt%) microgel ink. c) Light (top) and fluorescent (bottom) microscopy images of extruded ink composed of a mixture of equal volumes of NorHA (2 wt%, fluorescein isothiocyanate (FITC) labeled) and PEGDA (10 wt%, no label) microgels. d) Cross‐sectional diameters of extruded microgel ink filaments. *n* = 3 for each condition. NS: not significant. Scale bars: 200 µm.

Microgel inks were employed in two printing processes—a well‐established layer‐by‐layer extrusion method from a surface^1^, ^2^ and a gel‐in‐gel printing method of an ink into a support hydrogel,^43^ one of the emerging embedded 3D printing methods to deposit material into 3D space.^26^, ^29^, ^44^, ^45^, ^46^ In standard layer‐by‐layer printing (**Figure** **3**a and Movie S2, Supporting Information), the microgel ink was easily extruded onto a glass surface and then built into a lattice of up to four layers (Figure S6, Supporting Information). The features of the lattice—lines of filaments surrounding square voids—were maintained between the layers. As expected for an extrusion‐based process, modulating the extrusion rate, printing speed, or needle gauge corresponded to changes in printed filament diameters, from several hundred microns to over a millimeter (Figure S7, Supporting Information). To increase resolution for applications where this is needed, microgels with smaller diameters or smaller diameter nozzles might also be used.

**Figure 3:**
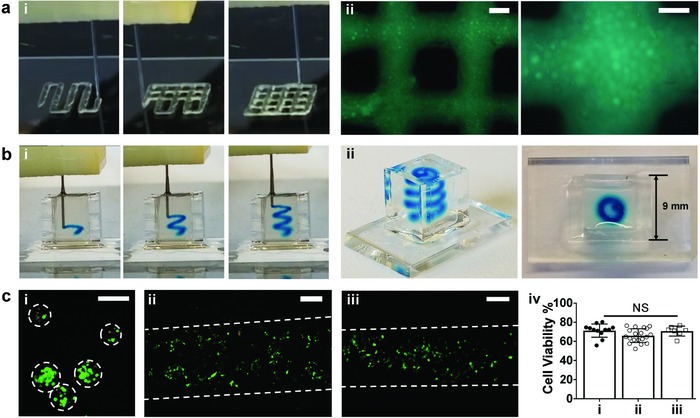
Jammed microgel ink printing on surfaces or within hydrogels. a) Images of (i) the extrusion‐based printing of microgel inks onto a glass surface and (ii) printed lattice structures from microgel inks containing FITC‐dextran. b) Images of (i) the printing of microgel inks (containing blue food coloring dye) in a spiral pattern within a shear‐thinning support hydrogel (transparent in the reservoir) and (ii) printed pattern viewed from the side (left) or top (right). c) Live/dead images of 3T3 fibroblasts encapsulated within microgels (i) after microgel fabrication or after jamming and extrusion (ii) onto a surface or (iii) within a shear‐thinning hydrogel, as well as (iv) quantification of cell viability for these conditions. Importantly, the jamming and printing process does not decrease cell viability. White dashed lines indicate approximate borders of microgels and all studies were performed with NorHA microgels. (i): *n* = 12; (ii): *n* = 19; (iii): *n* = 7 fields of view. NS: not significant. Scale bars in (a‐ii (left)): 500 µm, (a‐ii (right)): 200 µm, c): 200 µm.

As expected, mechanical forces disrupted printed structures (Figure S8, Supporting Information); thus, post‐cross‐linking was used to chemically link particles together. As an example, during the jamming process, additional cross‐linker (dithiothreitol) and photoinitiator (Irgacure 2959) were included, and ultraviolet (UV) light led to interparticle cross‐linking due to the presence of remaining unreacted norbornene groups. Printed and post‐cross‐linked lattice structures could subsequently be handled with forceps with no apparent loss in structural integrity (Movie S3, Supporting Information). Likewise, printed and post‐cross‐linked cuboid structures maintained their structure and dimensions for 7 days when placed in cell culture medium (Figure S8, Supporting Information). Furthermore, the introduction of interparticle bonds with post‐cross‐linking increased the compressive moduli of printed constructs (Figure S9, Supporting Information). This was to a value lower than hydrogels (no microgels) composed of the same formulation used to fabricate microgels, likely since the granular structure fails easier than materials with a uniform structure. Taken together, these results indicate that the printing of microgel inks using a layer‐by‐layer process is possible, as the jammed ink properties support printing and short‐term stability, while the post‐cross‐linking can be used to introduce long‐term stability.

Microgel inks were also deposited within shear‐thinning and self‐healing hydrogels (Figure S10, Supporting Information) in a gel‐in‐gel process, as has been done previously with other inks.^43^ NorHA microgel inks were extruded as mesoscale (<1 mm filament width) spiral patterns throughout macroscale space (≈1 cm) (Figure 3b and Movie S4, Supporting Information). The printed microgel ink resisted gravitational forces within the hydrogel to maintain the designed structure. With such an approach, inks printed into supports must generally resist internal forces that can result in breaking into droplets or being dragged by the print nozzle,^45^, ^47^ as well as external forces from the support such as dynamic rearrangements during self‐healing^48^ that might compress and cause flow of deposited inks.

In all variants of extrusion‐based 3D printing of soft materials for biofabrication, a central consideration is the viability of cells when embedded within the ink and during the printing process. Here, cells were encapsulated first within microgels and then jammed and printed. Cell viability was generally high (≈70%) after encapsulation within the microgels (Figure 3c‐i). This level of viability is consistent with a great number of studies where cells have been encapsulated within microgels.^34^, ^49^, ^50^ Importantly, with respect to the strengths of this method of formulating and printing materials, viability remained high (≈70%) after printing jammed microgel inks either onto a surface (Figure 3c‐ii) or into a support hydrogel (Figure 3c‐iii). These results indicate that cell viability is not influenced by either the jamming or printing processes, as the quantified viability was similar between the original microgels and after printing (Figure 3c‐iv). Here, the flow properties and elasticity of individual microgels of the inks likely work in tandem to protect cells by lowering forces and shielding cells from shear stresses during the printing process.

We believe the printing of jammed microgel inks opens up the possibility for the printing of a range of diverse materials and structures. To illustrate this, we utilized the gel‐in‐gel printing method of microgel inks into support hydrogels to print heterogeneous structures. Microgel inks were fabricated from microgels of various sizes (Figure S11, Supporting Information) and then printed into 3D support hydrogels as a checkerboard pattern (**Figure** **4**a,b), as discrete material pockets at the sub‐millimeter scale (Figure 4c), or as filaments of uniform or mixed compositions (Figure 4d). Further, microgel inks were used for the fabrication of microchannels within support hydrogels. To accomplish this, i) agarose‐based microgel inks were extruded into a support hydrogel, ii) the support hydrogel was stabilized by covalent cross‐linking, and iii) the agarose microgels were removed through dissolution with an increase in temperature to 37 °C and in the presence of agarase, leaving behind a perfusable channel within the stabilized support material (Figure S12 and Movie S5, Supporting Information).

**Figure 4:**
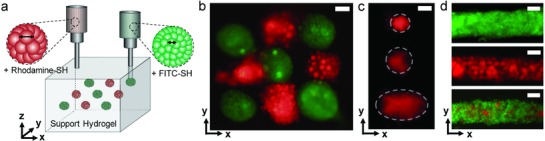
Printing of jammed NorHA microgel inks in diverse patterns within shear‐thinning support hydrogels. a) Scheme of printing an array of patterned ink depots within a support hydrogel from microgels incorporating either rhodamine (red, ≈100 µm) or FITC (green, ≈40 µm) dyes. Fluorescent images of b) an array of ink depots, c) ink depots of different sizes, and d) straight lines of individual or mixed microgel inks, all printed within a support hydrogel. White dotted circles in (c) indicate approximate borders of printed depots. Scale bars: 250 µm.

In conclusion, microgels were fabricated with a wide range of compositions and jammed into inks with remarkable flow and stabilization behaviors for extrusion printing. This approach decouples material printability from the printing technique, as microgels across various compositions can be used in ink formulation, enabling the printability of low‐viscosity materials that are useful but previously incompatible with printing. Jammed microgel inks could be printed either with layer‐by‐layer printing or within a support material, with secondary cross‐linking introduced when further stabilization is needed. Furthermore, microgel bioinks can be printed without damaging cells during the printing process, and highly heterogeneous structures are possible. The microgel approach permits control over cellular microenvironments through microgel design without the need for additives that may disrupt cell behavior. These unique materials offer exciting possibilities for new approaches to biofabrication and expand on the availability of inks for printing.

## Experimental Section

*Material Synthesis*: Polymers and peptides were synthesized as described in detail in previous protocols.^41^ Briefly, NorHA macromers were obtained by modifying the tetrabutylammonium salt of HA with 5‐norbornene‐2‐carboxylic acid (Nor) via esterification through di‐tert‐butyl dicarbonate (Boc_2_O) and 4‐dimethylaminopyridine (DMAP). ^1^H NMR (DMX 360 MHz, Bruker) was used to determine that ≈52% of HA repeat units of NorHA were modified with norbornene groups (Figure S2, Supporting Information). Standard fluorenylmethyloxycarbonyl (FMOC) chemistry was used to synthesize thiolated fluorescent peptides (GCDDD‐carboxyfluorescein, GCDDD‐Rhodamine B). Further details regarding material synthesis are included in Supporting Information.

*Microgel Fabrication and Jamming*: To fabricate microgels, mineral oil (Fisher Chemical) and NorHA/PEGDA/agarose solutions were separately pumped into the microfluidic devices and polymer droplets were created at the T‐junction of microchannels on devices (Figure S1, Supporting Information). While flowing through the tubing at outlets, the formed polymer droplets were cured by ultraviolet (UV, 320–390 nm, 15 mW cm^−2^, ≈30 s) or visible (400–500 nm, 200 W, 12 cm gap from the tubing, ≈12 s) light exposure or cooling (4 °C). To fabricate cell‐laden microgels, the NorHA solution containing NIH 3T3 fibroblasts at a density of 10 million cells per milliliter was flowed through the device and visible light was used to cross‐link the microgels. Microgels collected with mineral oil were suspended in phosphate buffered saline (PBS) and centrifuged, and the oil layer was aspirated. Microgels were further rinsed in pure PBS and jammed by vacuum‐driven filtration (Steriflip, 0.22 µm pores, Millipore). Further details regarding the fabrication process of microgels are included in Supporting Information.

*Rheological Characterization*: Rheological properties of jammed microgel inks were measured using a rheometer (AR2000) with a 20 mm parallel plate geometry, 1 mm gap (for NorHA, PEGDA microgel inks) or 200 µm gap (for agarose microgel ink) at 25 °C. To explore shear‐thinning properties, viscosity was measured with a continuously ramped shear rate (from 0 to 50 s^−1^). Strain sweeps (from 0.037 to 1000%, 1 Hz) and oscillatory frequency sweeps (from 0.01 to 100 Hz, 1% strain) were performed to examine ink properties. For shear recovery experiments, low (1%) and high (500%) strains were periodically applied at 1 Hz.

*3D Printing*: A modified Revolution XL printer (Quintessential Universal Building Device, Inc.) was used for 3D printing as previously described.^43^ Briefly, a funnel containing jammed microgel inks was placed on top of the syringe and centrifuged at 1000 × *g* for 3 min to load the inks into the syringe. A syringe plunger, actuated using a stepper motor, was used to extrude jammed microgels through a 25 G needle. Printing paths were generated using G‐code commands (Slic3r) that were implemented via Repetier hardware control. To analyze the influence of printing parameters on cross‐sectional diameters of extruded filaments, extrusion rates and printing speeds were controlled through G‐code commands, and different size needles were used for printing (Figure S7, Supporting Information). Cross‐sectional diameters of extruded filaments were assessed via Image J. To print lattice and spiral structures, printing speeds of 20 and 10 mm min^−1^ were used, respectively. Printing was performed either onto a surface or throughout a support hydrogel. When the ink was printed on a surface, a small gap between the nozzle and the surface was maintained to permit the deposition of the ink (as described in detail in the Supporting Information).

*Statistical Analysis*: Data is presented as mean ± standard deviation. Statistical analysis was conducted using ANOVA and a Tukey's post hoc comparison.

## Conflict of Interest

The authors declare no conflict of interest.

## Supporting information

Supplementary

Supplementary

Supplementary

Supplementary

Supplementary

Supplementary
