# Supplementary material for: Jammed Microgel Inks for 3D Printing Applications
Source: Adv Sci (Weinh). 2018 Oct 24;6(1):1801076. doi: 10.1002/advs.201801076 (PMC6325587; doi:10.1002/advs.201801076)
Supplement: Supplementary file 1 — Supplementary [file ADVS-6-1801076-s002.pdf]

Copyright WILEY-VCH Verlag GmbH & Co. KGaA, 69469 Weinheim, Germany, 2018.

ADVANCED SCIENCE Open Access

Supporting Information

for *Adv. Sci.,* DOI: 10.1002/advs.201801076

Jammed Microgel Inks for 3D Printing Applications

*Christopher B. Highley, Kwang Hoon Song, Andrew C. Daly, and Jason A. Burdick**

Copyright WILEY-VCH Verlag GmbH & Co. KGaA, 69469 Weinheim, Germany, 2016.

Supporting Information

Jammed Microgel Inks for 3D Printing Applications

*Christopher B. Highley[#], Kwang Hoon Song[#], Andrew C. Daly, Jason A. Burdick**

[#]Authors contributed equally
*Corresponding author. Telephone: 215-898-8537; E-mail: burdick2@seas.upenn.edu

Microfluidic Device Fabrication: Molds for microfluidic devices were fabricated by conventional photolithography[1] or stereolithography (Material: MicroFine Green, Proto Labs). The devices were cast using polydimethylsiloxane (PDMS), punched with 1 mm-diameter biopsy punches (Integra Miltex, Kai Medical) to form inlets and outlets, and attached to PDMS substrates via plasma treatment. Next, 18 G blunt needles (McMaster-Carr) were inserted into the inlets and outlets of the devices, and tubing (ID: 0.79 mm, OD: 2.38 mm, ABW00001, Saint-Gobain) was connected to the needles.

Microgel Fabrication and Jamming: To fabricate microgels, microchannels of devices (Figure S1) were first primed with mineral oil (Fisher Chemical) supplemented with 2 % span 80 at the flow rate of 30 µl min^{-1}. A polymer solution consisting of 2 wt% NorHA, 7.8 mM thiolated crosslinkers (DL-dithiothreitol, DTT) and 0.05 wt% Irgacure 2959 (Ciba) was then introduced at 3 µl min^{-1} and droplets of polymers were generated at the merging points of both fluids (Figure S1). While flowing through the outlet tubing, the polymer droplets were crosslinked by exposure to ultraviolet light (UV, 320 - 390 nm, 15 mW cm^{-2}, ~30 s). When visible light (400 - 500 nm, 200 W, 12 cm-gap from the tubing, ~12 s) was exposed to cure droplets (for inks used in Figure 3), a solution consisting of 3 wt% NorHA, 10 mM DTT and 0.05 wt% lithium phenyl-2,4,6-trimethylbenzoylphosphinate (LAP) was flowed through devices at the same flow rate. For fabrication of PEGDA microgels, a solution consisting of

10 wt% PEGDA (6 kDa) and 0.1 wt% Irgacure 2959 was flowed through devices, and the generated droplets were crosslinked by UV exposure (320 - 390 nm, 15 mW cm^{-2}, ~30 s). For fabrication of agarose microgels, a solution of 1 wt% ultra low melting temperature agarose (Lonza) was flowed through devices, and the droplets were crosslinked by cooling outlet tubing and a collecting reservoir (4 ºC). To generate cell-containing microgels, NIH 3T3 fibroblasts were introduced into the 3 wt% NorHA solution (with 2 mM thiolated RGD added, GenScript) at a density of 10 million cells ml^{-1} and visible light (400 - 500 nm, 200 W, 12 cm-gap from the tubing, ~12 s) was introduced to crosslink the microgels. Microgels were suspended in PBS and centrifuged at 1,000 G for 5 min, and the oil layer on top was aspirated. The rinsed microgels in pure PBS were jammed by vacuum-driven filtration (Steriflip, 0.22 μm-pores, Millipore) to use as inks for 3D printing. Image J was used to quantify diameters and roundness of microgels.

Support Hydrogel Synthesis and Formation: All chemicals were purchased from Sigma-Aldrich, unless indicated otherwise. AdNorHA[2] and CDHA[3] used throughout the experiments were synthesized, as described previously. Briefly, AdNorHA was obtained by functionalizing the tetrabutylammonium salt of HA (HA-TBA) with 1-adamantane acetic acid (Ad) and 5-norbornene-2-carboxylic acid (Nor) simultaneously via esterification through ditert-butyl dicarbonate ($B_{OC}2O$)/4-dimethylaminopyridine (DMAP). ^{1}H NMR was used to determine that ~10.8 % and ~30.5 % of HA repeat units were modified with Ad and Nor, respectively (Figure S10a). For CDHA, HA-TBA was functionalized with aminated *β*-cyclodextrin (*β*-CD, TCI America) via amidation through benzotriazole-1-yl-oxy-tris-(dimethylamino)-phosphonium hexafluorophosphate (BOP). ^{1}H NMR was used to confirm that ~19.8 % of HA repeat units were modified with *β*-CD (Figure S10b). To form support hydrogels, AdNorHA and CDHA macromers (final polymer concentration: 3 wt%, Ad and CD ratio: 1:1) were separately dissolved in PBS containing 0.05 wt% Irgacure 2959 and 10 mM DTT. Next, the two macromer solutions were mixed thoroughly and centrifuged at

>10,000 G to remove entrapped air. The self-assembled hydrogels were then loaded into custom PDMS molds to enable printing of the jammed microgel inks into the support material.

Cell Culture and Live Dead Staining: NIH 3T3 fibroblasts were expanded in growth medium (Minimum essential medium, 10% fetal bovine serum (FBS), 1% L-glutamine, and 1% penicillin-streptomycin). To assess post-printing cell viability, the printed cell-laden microgel structures were gently immersed in calcein-AM/ethidium homodimer solution for 60 min without post-crosslinking processes. Fluorescence microscopy (Olympus BX51) was used to image the stained cell-laden structures and Image J was used to quantify cell viability.

Pressure Measurement during the Extrusion of Inks: 3 experimental groups were loaded into 1 mL tuberculin syringes fitted with 6.35 mm-length 25 G needles. These groups were: jammed microgel ink, crosslinked polymer, and polymer solution before crosslinking. Syringes were loaded onto an Instron fitted with a 100 N-load cell and plungers were depressed by the Instron at a constant rate of 1 mm s^{-1}. The resulting forces measured were converted to pressures based on the plunger diameter, and reported with respect to net material extruded at the constant extrusion rate.

Stability Analysis of 3D Printed Structures: 3D printed cuboid structures were immersed in cell culture media consisting of Minimum Essential Medium (MEM, Gibco) with 20 % fetal bovine serum and 1 % penicillin/streptomycin without or with post-crosslinking. For post-crosslinking, acellular NorHA microgels were pre-soaked in PBS solution containing 5 mM DTT and 0.05 wt% Irgacure 2955 for 5 min prior to jamming and use as inks. After printing, the structures were exposed to UV light (320 nm – 390 nm, 10 mw cm^{-2}, 5 min) to stabilize the samples. Cell culture media was changed on day 3. Edge lengths of post-crosslinked cuboid structures were analyzed via Image J on day 0, 3, and 7.

Measurement of Elastic Moduli of 3D Printed Structures: The elastic moduli of 3D printed structures were measured using a Dynamic Mechanical Analyzer (DMAQ800, TA Instruments). A force ramp of 0.5 N min^{-1} to max 15 N was applied to the printed sample to

obtain the stress versus strain curve, and the elastic modulus was determined as the slope between 10 – 25 % strain.

Supporting References

[1] T. L. Rapp, C. B. Highley, B. C. Manor, J. A. Burdick, I. J. Dmochowski, *Chem. Eur. J.* **2018**, *24*, 2328.

[2] J. E. Mealy, J. J. Chung, H.-H Jeong, D. Issadore, D. Lee, P. Atluri, J. A. Burdick, *Adv. Mater*. **2018**, 1705912.

[3] C. Loebel, C. B. Rodell, M. H. Chen, J. A. Burdick, *Nat. Protoc*. **2017**, *12*, 1521.

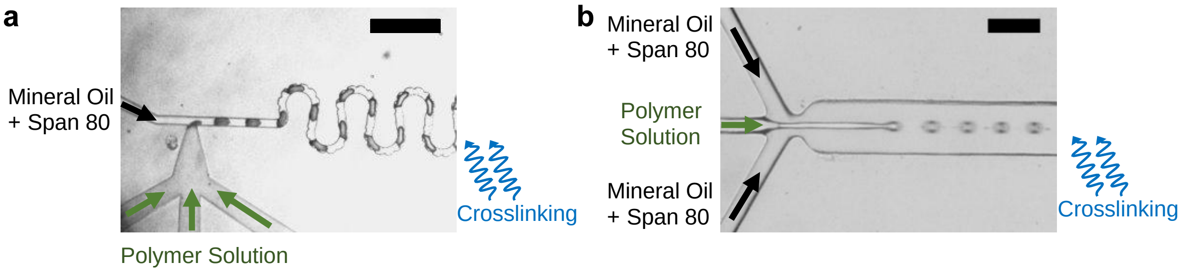

Figure S1. Microfluidic devices for microgel fabrication. a, b) Light microscopy images of microfluidic devices generating aqueous polymer droplets via water-in-oil emulsions. The polymer droplets were crosslinked (via light or cooling) at the outlet of the microfluidic devices to form microgels that were further washed from the oil. Green and black arrows indicate flow directions of polymer solution and mineral oil with 2% span 80, respectively. Scale bars in (a): 200 µm, (b): 300 µm.

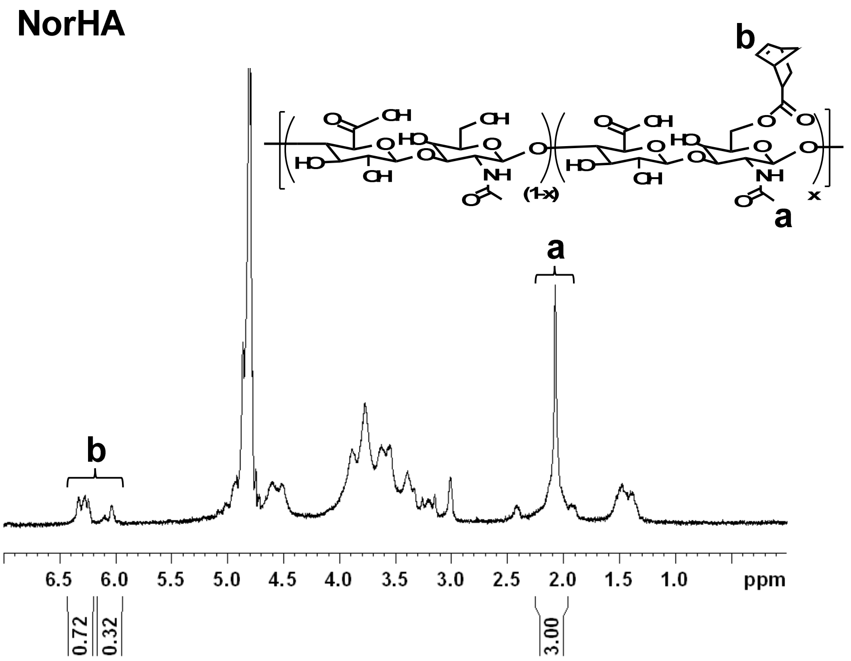

Figure S2. ^{1}H NMR spectrum of synthesized norbornene-modified hyaluronic acid (NorHA) polymers. The degree of modification (~52.0 %) was determined by integration of norbornene vinyl protons (δ = 5.94 - 6.17, 1 H and δ = 6.20 - 6.44, 1 H), relative to HA methyl singlet (δ = 2.1, 3 H).

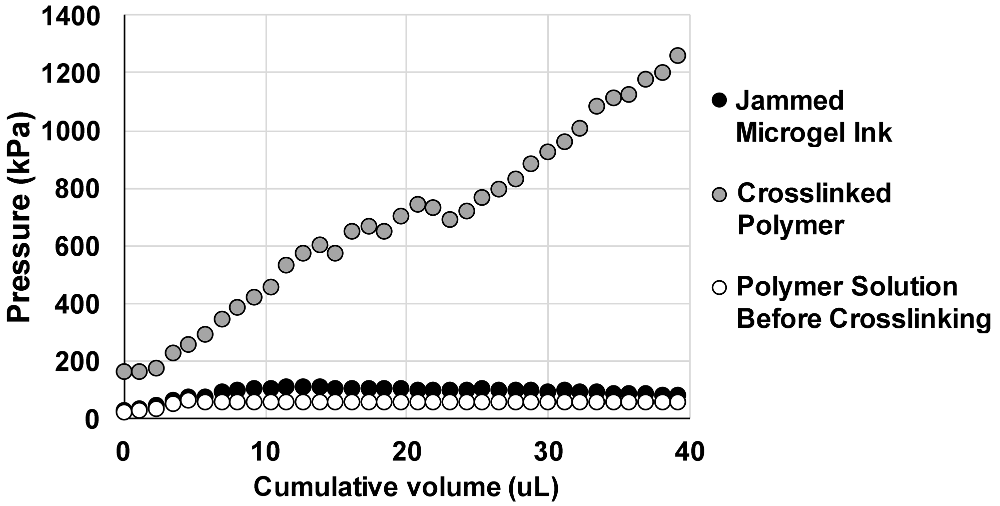

Figure S3. Extrusion forces for microgel inks. Measurement of pressures during the extrusion of jammed microgel ink, crosslinked polymer, and polymer solution before crosslinking at the rate of 0.72 ml min^{-1}. The polymer formulation (2 wt% NorHA, 7.8 mM thiolated crosslinker (DTT) and 0.05 w% Irgacure 2959) was identical for all conditions. UV light (320 nm – 390 nm, 15 mw cm^{-2}) was used for crosslinking of the polymer solution for 1 min.

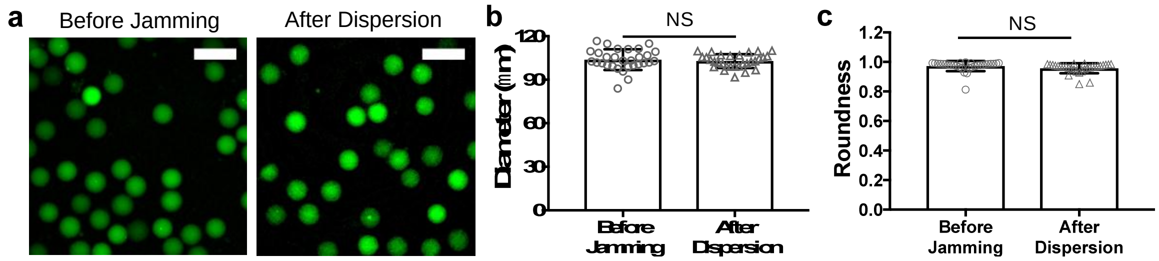

Figure S4. Characterization of NorHA microgels after fabrication and after the printing process. a) Fluorescent images and analysis of (b) diameter and (c) roundness of NorHA microgels, before the jamming process and after dispersion of printed microgel inks. Roundness indicates: 4 x [Area] x (π x [Major axis]^2)$^{-1}$. n = 30 for each condition. NS: not significant. Scale bars: 200 µm.

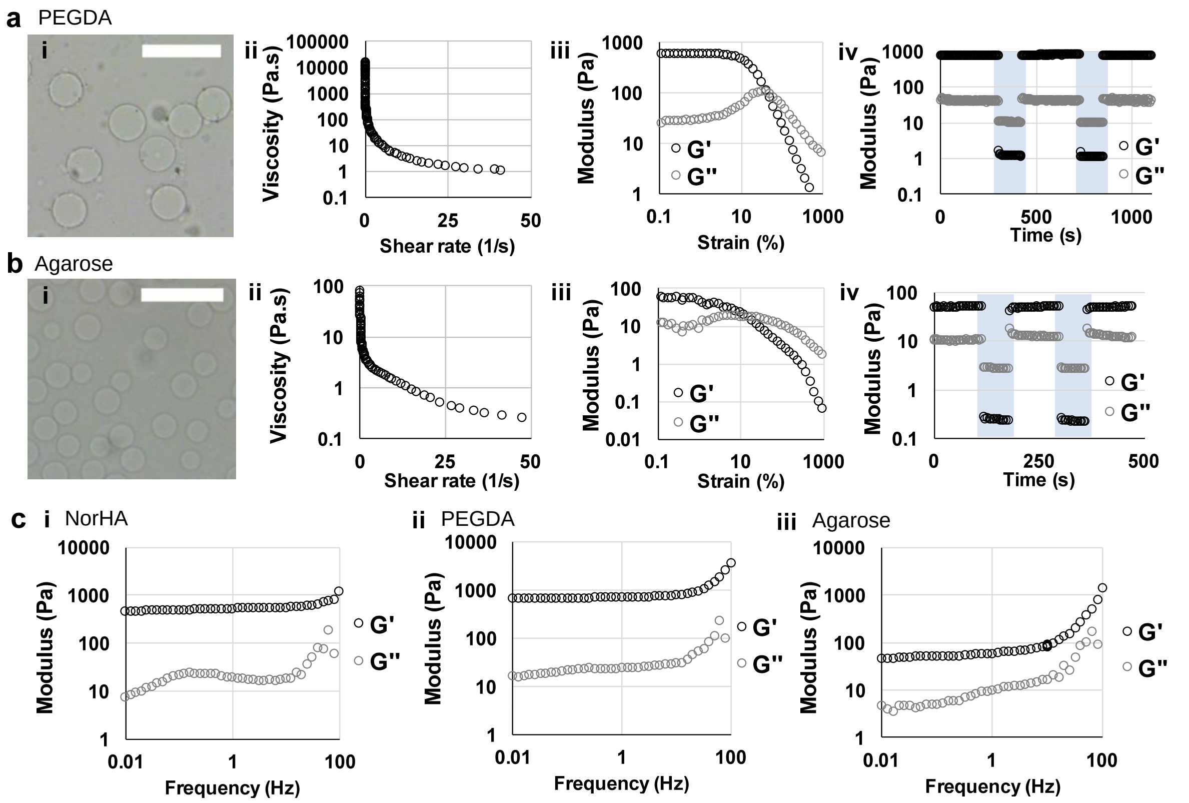

Figure S5. Characterization of NorHA (2 wt%), PEGDA (10 wt%) and agarose (1 wt%) microgel inks. Light microscopy images of (a-i) PEGDA and (b-i) agarose microgels. Rheological characterization of jammed (a-ii,iii,iv) PEGDA and (b-ii,iii,iv) agarose microgel inks. Panels indicate: (ii) decreased viscosity with increasing shear rate (0 to 50 s^{-1}), (iii) shear-yielding with increase in strain (0.037 to 1000 %, 1 Hz), and (iv) shear-thinning and self-healing through low (unshaded, 1 % strain, 1 Hz) and high (shaded, 500 % strain, 1 Hz) strain cycles. Representative oscillatory frequency sweeps (0.01 to 100 Hz, 1 % strain) of (c-i) NorHA, (c-ii) PEGDA and (c-iii) agarose microgel inks. Scale bars: 200 µm.

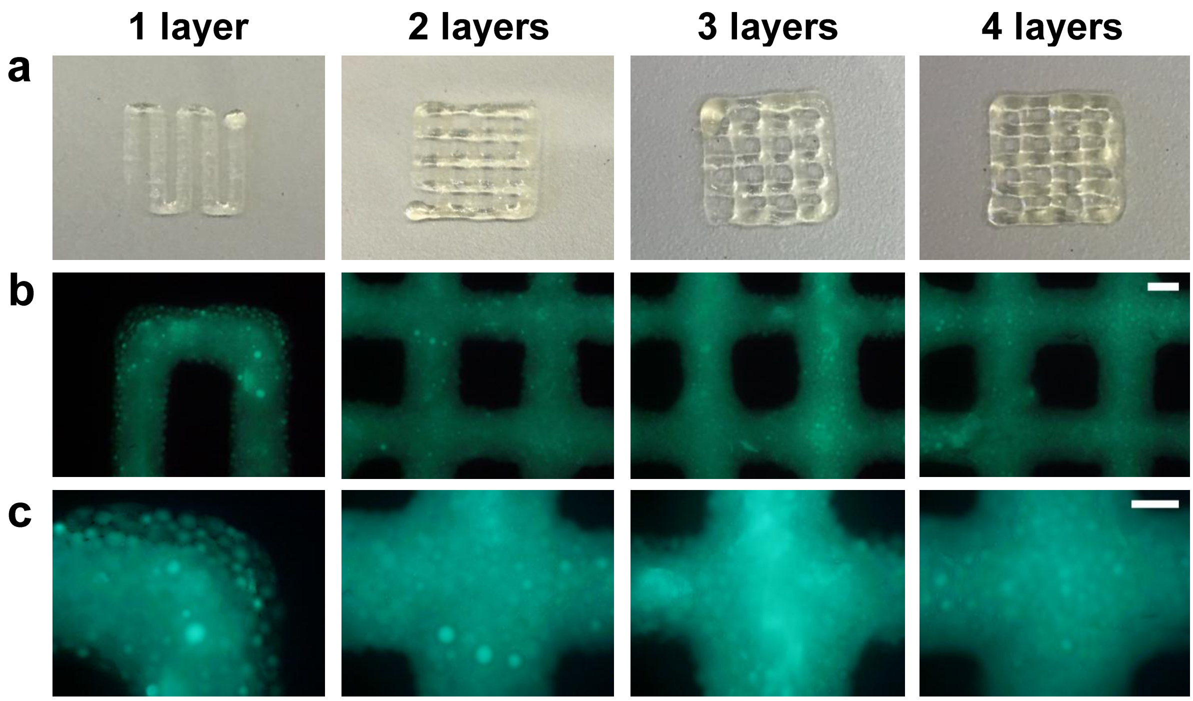

Figure S6. Images of lattice structures printed through a 25 G needle on a glass surface with jammed NorHA microgel inks. a) Macroscopic image and (b,c) fluorescent images of 1-4 layers of lattice structures printed with inks containing FITC-dextran. Scale bars in (b): 500 μm, (c): 200 μm.

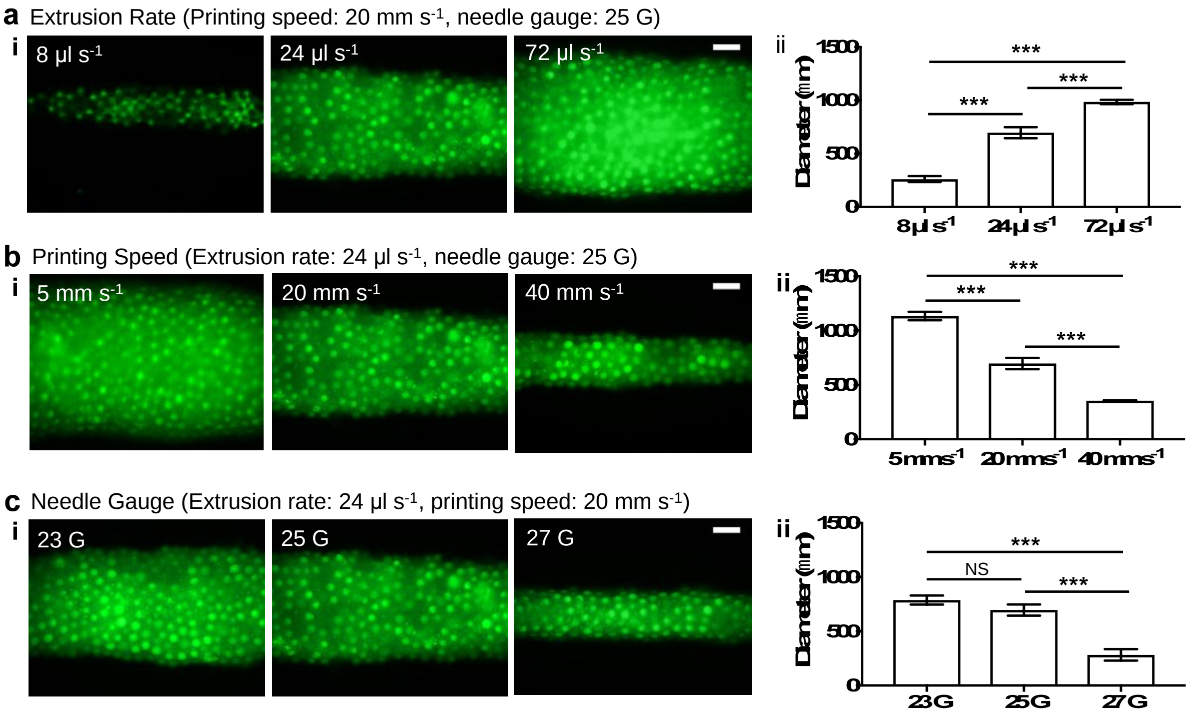

Figure S7. Influence of printing parameters (extrusion rate, printing speed, and needle gauge) on cross-sectional diameters of extruded filaments with jammed NorHA microgel ink. (i) Fluorescent images and (ii) cross-sectional diameters of extruded filaments printed with varied: a) extrusion rates of 8, 24, 72 μl s^{-1} (printing speed: 20 mm s^{-1}, needle gauge: 25 G), b) printing speeds of 5, 20, 40 mm s^{-1} (extrusion rate: 24 μl s^{-1}, needle gauge: 25 G), and c) needle gauges of 23, 25, 27 G (extrusion rate: 24 μl s^{-1}, printing speed: 20 mm s^{-1}). $n = 3$ for each condition. ***$p < 0.001$, NS: not significant. Scale bars: 200 μm.

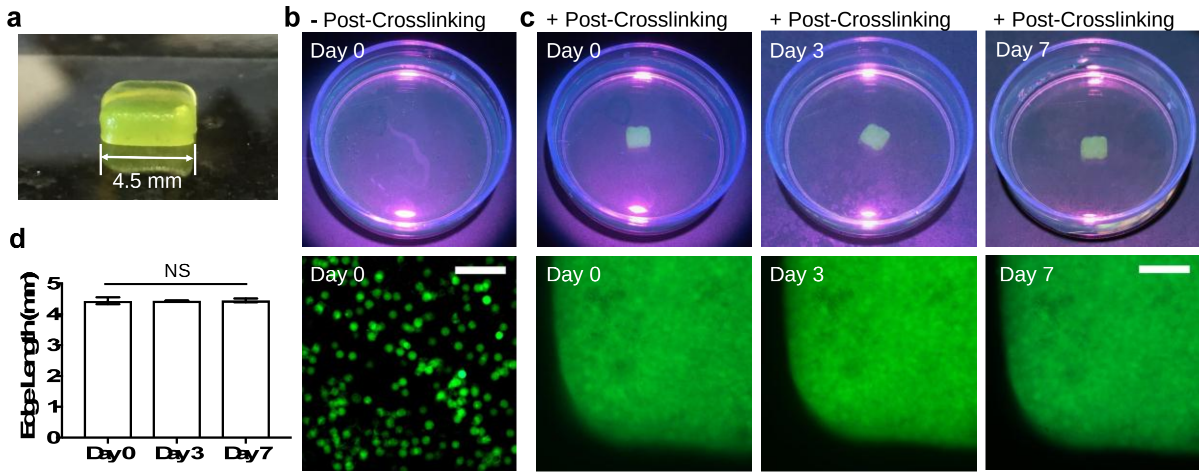

Figure S8. Stability of cuboid structures printed with jammed NorHA microgel ink. a) Macroscopic image of cuboid structure printed with a jammed NorHA microgel ink. Macroscopic (top) and fluorescent (bottom) images of the printed samples after immersion in cell culture media either (b) without (-) post-crosslinking or (c) with (+) post-crosslinking. For post-crosslinking, samples were exposed to UV light (320 nm – 390 nm, 10 mw cm^{-2}) for 5 min after printing. d) Edge length quantification of the printed and post-crosslinked samples after immersion in cell culture media on day 0, 3, and 7. n = 3 for each condition. NS: not significant. Scale bars: 500 µm.

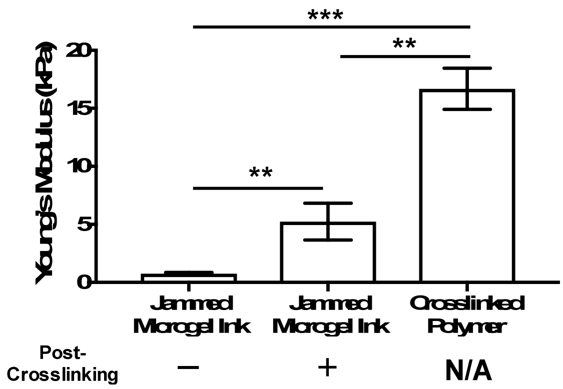

Figure S9. Mechanical properties of 3D printed structures from jammed microgel inks. Compressive young's moduli of 3D printed structures from jammed microgels either without (-) or with (+) post-crosslinking or of hydrogels fabricated directly from the pre-polymer solution (no microgels). The pre-polymer formulation (2 wt% NorHA, 7.8 mM thiolated crosslinker (DTT) and 0.05 w% Irgacure 2959) was identical for all conditions, either for microgel fabrication or direct hydrogel formation (UV light: 320 nm – 390 nm, 10 mw cm^{-2}, 1 min). For post-crosslinking, the printed samples were exposed to UV light (320 nm – 390 nm, 10 mw cm^{-2}) for an additional 5 min. n = 3 for each condition. $^{**}p < 0.01$, $^{***}p < 0.001$.

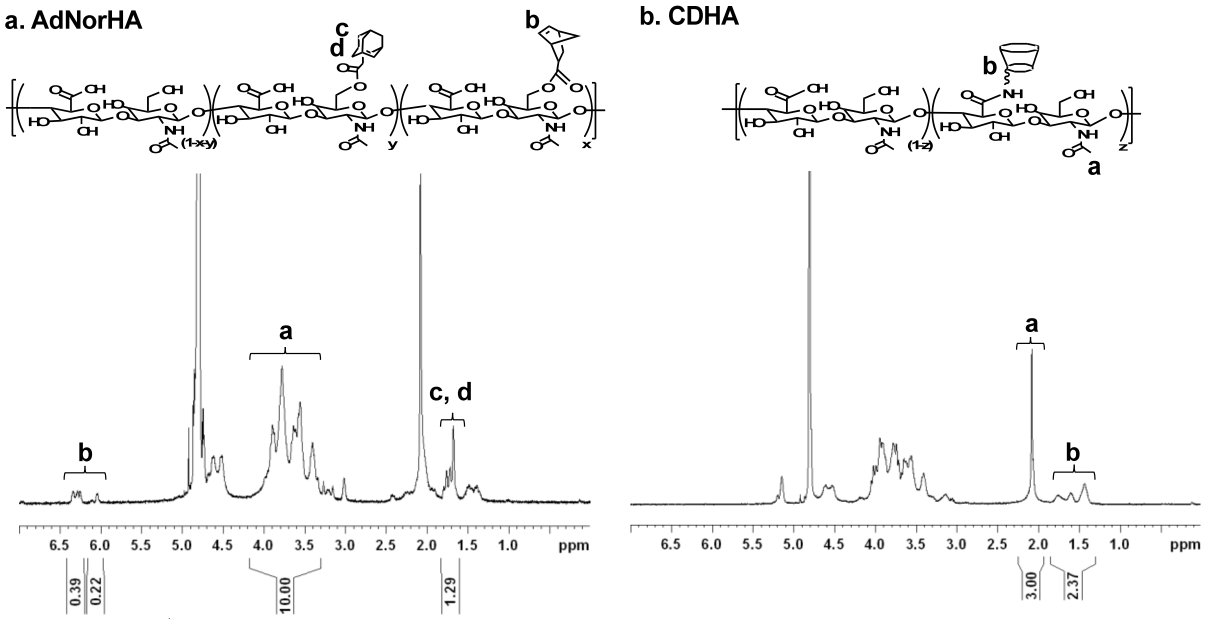

Figure S10. ^{1}H NMR spectra of synthesized HA polymers for formation of shear-thinning support hydrogels. a) Adamantane- and norbornene-modified HA (AdNorHA): The degrees of modifications (~10.8 %, ~30.5 %, respectively) were determined by integration of adamantane ethyl multiplet (δ = 1.60 - 1.83, 12 H) and norbornene vinyl protons (δ = 5.97 - 6.16, 1 H and δ = 6.21 - 6.42, 1 H), respectively, relative to HA disaccharides (δ = 3.30 - 4.18, 10 H). b) *β*-cyclodextrin-modified HA (CDHA): The degree of modification (~19.8 %) was determined by integration of hexane linker (δ = 1.30 - 1.86, 12 H), relative to HA methyl singlet (δ = 2.1, 3 H).

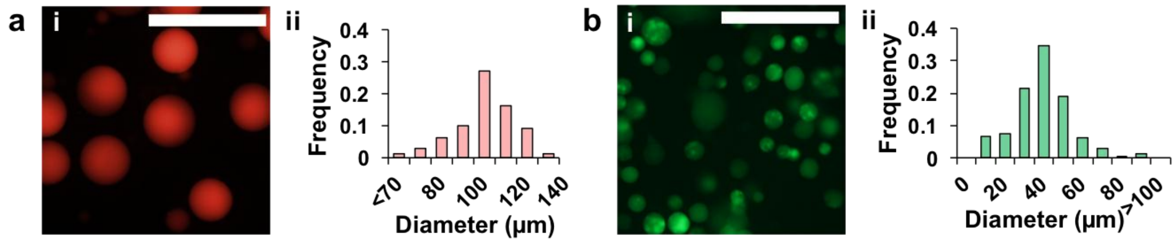

Figure S11. Characterization of microgels for printing into 3D support hydrogels. (i) Fluorescent images and (ii) quantification of size distributions of (a) rhodamine-thiol- and (b) FITC-thiol-tethered NorHA microgels. Scale bars: 300 μm.

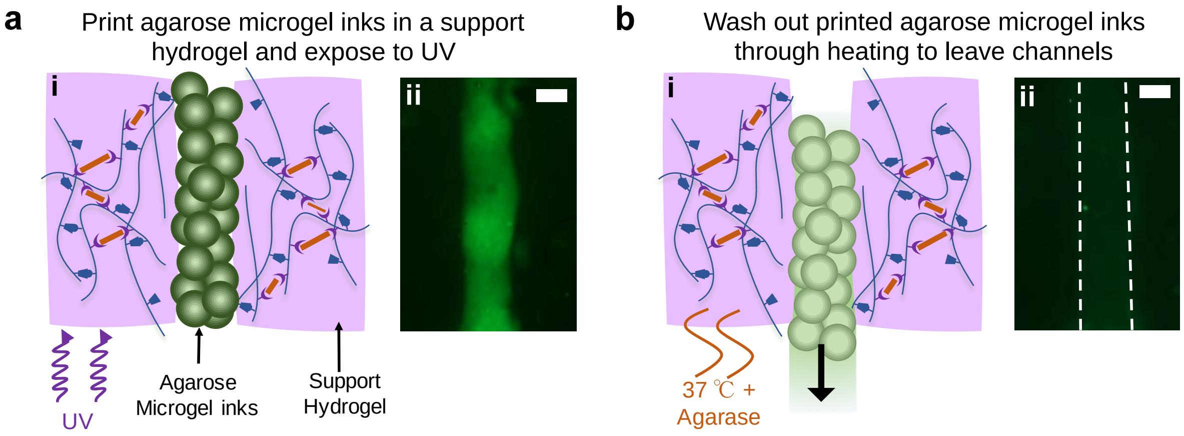

Figure S12. Fabrication of microchannels in shear-thinning support hydrogels. a-i) After printing agarose microgel inks in a support hydrogel, they were exposed to UV to crosslink the support hydrogel. a-ii) Fluorescent image of a printed straight line of agarose microgel inks containing FITC-dextran. b-i) Heating (37 ℃) and agarase were applied to solubilize printed inks. By washing out the solubilized inks, an open microchannel was obtained in the support hydrogel. b-ii) Fluorescent image of the support hydrogel, after washing out the printed ink. White dashed lines indicate approximate borders of microchannel. Scale bars: 500 µm.

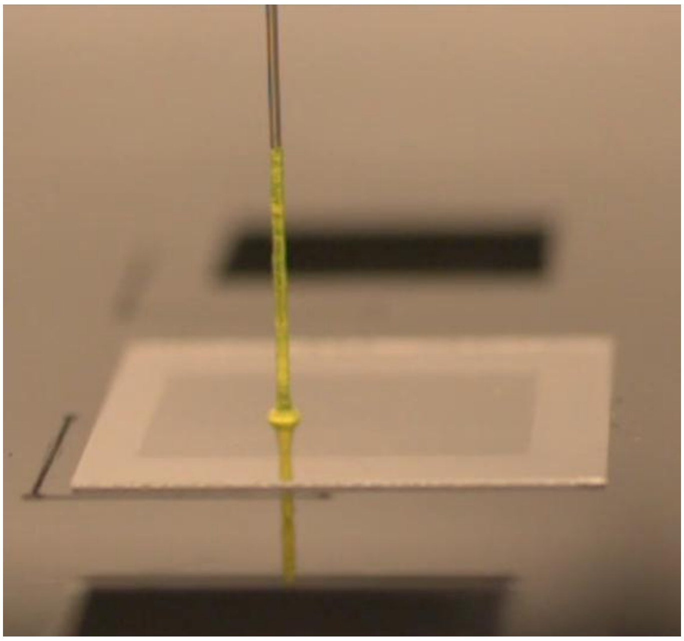

Movie S1. Extrusion of jammed NorHA microgel inks through a 25 G needle (OD: 0.51 mm, ID: 0.26 mm) and translation of the needle during the extrusion to show integrity and elasticity of inks.

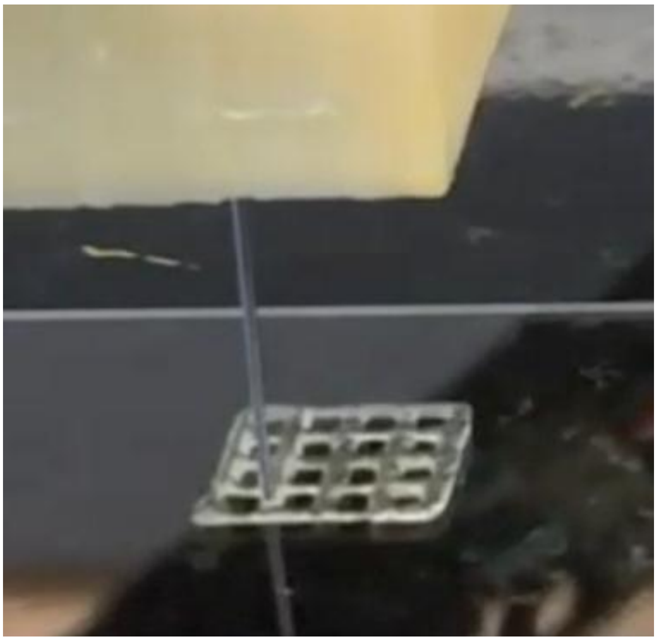

Movie S2. Printing of lattice structures on a glass surface with jammed NorHA microgel inks. Printing speed: 20 mm min^{-1}.

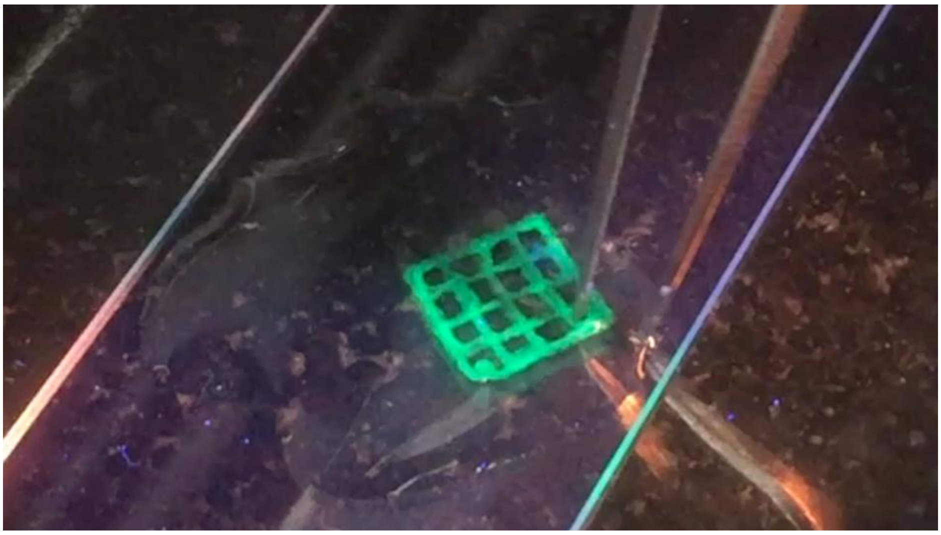

Movie S3. Stabilized lattice structures that were printed on a glass surface. After printing with jammed NorHA microgel inks containing unbound dithiol crosslinker (DTT, 10 mM) and photoinitiator (0.05 wt%, I2959), the printed structures were exposed to UV (320 - 390 nm).

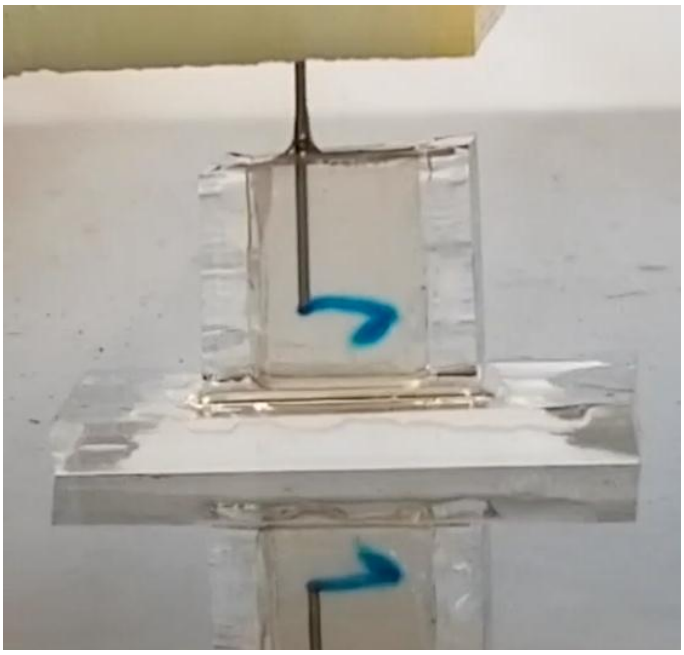

Movie S4. Printing of spiral structures into a shear-thinning support hydrogel (transparent in the reservoir) with jammed NorHA microgel inks containing blue food coloring dye. Printing speed: 10 mm min^{-1}.

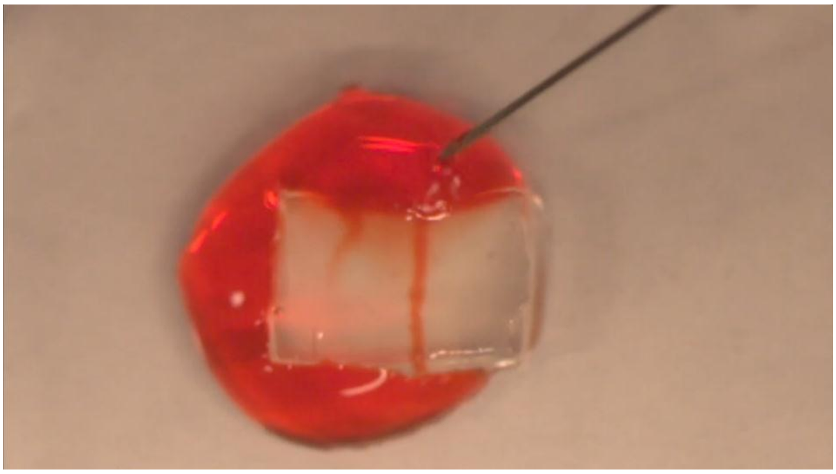

Movie S5. Repeated perfusion of food coloring dyes through a microchannel fabricated in a shear-thinning support hydrogel construct.
